# Supplementary material for: Patterns of Intron Gain and Loss in Fungi
Source: PLoS Biol. 2004 Nov 30;2(12):e422. doi: 10.1371/journal.pbio.0020422 (PMC532390; doi:10.1371/journal.pbio.0020422)
Supplement: Table S1 — Also available at http://genes.mit.edu/NielsenEtAl/. (4.3 MB ZIP). [file pbio.0020422.st001.zip › NielsenEtAl/html/377.html]

AN5588.1.NCU03359.1.MG03926.1.FG01475.1


```
 CLUSTAL W (1.82) Multiple Sequence Alignments - Introns Inserted


Sequence 1: MG03926.1	748 aa
Sequence 2: FG01475.1	790 aa
Sequence 3: NCU03359.1	738 aa
Sequence 4: AN5588.1	802 aa
Alignment Length: 831 aa
Number Identitical Residues: 280 aa
Alignment Score (without introns) 13636


MG03926.1 	----------~---------------~----------------MAPHA~SA---LP0NMA
NCU03359.1	----------~---------------~---------------------~-------~---
FG01475.1 	----------~-----------MVLP0ARRSGSSKEAIRENKAAEPHQ0AAELRLP~EFL
AN5588.1  	MASQLPILKP0NVSASGSWPSMSKFL~KSPLHRTGQSFATIGSAIQDS~RRRLHTE0TFA
          	 ::. .  .. . :::.: .:            : .:     :                 

MG03926.1 	AAASIAGSLATRRAIFLPNSASRIIRSTASESLIANSIIRTYSALQSPSRLFRNAP----
NCU03359.1	--------MSSRQLAMANPLFRRSFSALMSRPLGTVNTLRSMSTHQP-GRIPSFFR----
FG01475.1 	QKVDLTSFSRSRFSAMAPMTAQATISNMGRVGLHAANPILRHPYFQPRASTPSSFM----
AN5588.1  	RKSTPSEVVAFGVYSAPVHRLPRSLLSSKRNPLSSSWTASDSGDLESSIFTPSSFAGRHC
          	     :                  :       * :          :.         .  .

MG03926.1 	----LGASTGRHIQAASGSNLQFP---HQRLFMS--GISRTLLAQREETANRNPSSASAQ
NCU03359.1	----SPVHSSLGFT------------LQVRSFGNG-GLSHNLLAAREAAANQFPTSAGAQ
FG01475.1 	----RSFSTSSGILSSRGISPLGPFSVQRRAFGSSNGISRNQLATLEESANRNPGNANAQ
AN5588.1  	HPLASKTYSTLAPPLNRTRNVLIPSTLQQQRFIFG-GPSHSLLAQKEKTANNNPSSANAQ
          	 . :    :          .   . : : : *  . * *:. **  * :**. * .*.**

MG03926.1 	NAFYQTLLKANMPAIIVERHQSGHFASNDATREVYNRARAMLEG--SQGASNSVAANGEF
NCU03359.1	YAFYQALLKANMPAIIIERYQSGRFATNEQVDQIYQQALAMSTGQPYTPANNTVDNNGYH
FG01475.1 	NAFYQLLLRANMPAILVERYQSGRFATSPATNDAYQRALAMLGASATQAANAPGATNGNF
AN5588.1  	NAFYQALLRANMPAIVIERYRSGHFSSNAVSEQIYLKALERVGGGVSAPAAN--LNQGLR
          	 **** **:******::**::**:*::.    : * :*     .     *      :*  

MG03926.1 	VE--DTSRLRAVGQAVAARSMGGNISSAR-----ETANGPIRVVVDESRGSQIFRWVKFV
NCU03359.1	PSGFTASQIHAAGTAAAAQHTGGNMAMVKPIA-AGAKTGPLHIVVDESFGSSALRWVKFL
FG01475.1 	GR--TQWPTYEQGIANAAVTGAANAGNPM-----GHKGEPIHVIVQESTRSTIFRWVKFL
AN5588.1  	SD-----QIQAVGQAVAAQNQGGQIGISSKQSGTGAKEAPLYVVVEESLGSAVFRWVKFI
          	            * * **   ..: .     :.:     *: ::*:**  *  :*****:

MG03926.1 	AYFAFVAYVSMVVLGIVFDMFTSVNKK-MSTQKHMEVKADKQKTRFKDVHGCEEAKEELQ
NCU03359.1	MWFTLFTYLSMVVITMVFEGLSSIKRP-GGKLEASEVKPENQKARFADVHGCDEAKEELQ
FG01475.1 	AIFIVTTYLCFALVTIAIEAFSTFRRGGPSSKQDSEVKAEKQNTRFQDVHGCDEAKEELQ
AN5588.1  	VLFCAFAYASMIVLSIVLETTGVLKNIKGPHSN--EAQPEHQTVRFSDVHGCDEAKDELQ
          	  *   :* .: :: :.::    ...      :  *.:.::*..** *****:***:***

MG03926.1 	DLVEFLKNPDKFSSLGGKLPKGVLLVGPPGTGKTLLARAVAGEAGVPFFYMSGSEFEEVF
NCU03359.1	ELIDFLRNPEKYSTLGGKLPKGVLLVGPPGTGKTLLARAVAGEAGVPFFNMSGSEFEEVY
FG01475.1 	EVVEFLKNPEKFSDLGAKLPKGVLLVGPPGTGKTLLARAVAGEAGVPFFYMSGSEFDEIF
AN5588.1  	ELVEFLLNPERFSSLGGKLPKGVLLVGPPGTGKTLLARAVAGEAGVPFFYMSGSEFDEVY
          	::::** **:::* **.******************************** ******:*::

MG03926.1 	VGVGARRVRDLFTAAKTASPAIVFIDELDAIGGKRNAKDPSYAKQTLNQLLTELDGFEQD
NCU03359.1	VGVGAKRVRDLFAAAKAKAPSIVFIDELDAIGGRRNSRDATYVRQTLNQLLTELDGFEQN
FG01475.1 	VGVGAKRVRELFTAAKNKSPAIVFIDELDAIGGKRNPRDQAHAKQTLNQLLTELDGFDQD
AN5588.1  	VGVGAKRVRELFNQARSKSPAIIFIDELDAIGAKRNERDAAYVKQTLNQLLTELDGFSQS
          	*****:***:**  *:  :*:*:*********.:** :* ::.:*************.*.

MG03926.1 	SGVIVIAATNFPRSLDKALTRPGRFDRHVQVDLPDIRGRIAILKHHATKIKADDNIDYEA
NCU03359.1	SGVIIIGATNFPESLDKALTRPGRFDRNVVVSLPDVRGRMAILQHHAKRIKAAADVNLEA
FG01475.1 	SKIIIIGATNLPKMLDKALTRPGRFDRHVNVDLPDVRGRIAILKHHAKKIKVSPDVDLEA
AN5588.1  	TGVIILAATNYPELLDKALTRPGRFDRKVVVDLPDVRGRMDILKHHMKNVQISTDVDVAV
          	: :*::.*** *. *************:* *.***:***: **:** ..::   :::  .

MG03926.1 	IARSTPGLSGAELESIVNQAAIHASKTRKTIVSTKDFDWAKDKVIMGAERRSMVISPKEK
NCU03359.1	IASRTSGLSGAELENIVNQAAIHASKLKAQAVTQKDFEWAKDKVIMGAEKRSMVITAKEK
FG01475.1 	IAARCPGQSGAELENMLNVAALRASRAKASFVSKQDMEWAYDRVTMGSERKSMVITEKEK
AN5588.1  	IARGTSGFSGADLENLVNQAAIFASRNKQSKVTPKDFDYAKDKIIMGAEARSRIIQDKDK
          	**   .* ***:**.::* **: **: :   *: :*:::* *:: **:* :* :*  *:*

MG03926.1 	EMTAYHEAGHALITLYTQDSPNKLYKVTILPRGPSLGHTAYLPEMDKYSYTVKDYKAQID
NCU03359.1	EMTAYHEAGHALVGYYAKDSASSLYKVTILPRGQTLGHTAYLPEMDKHSFTVRDYLGMID
FG01475.1 	EMTAYHEAGHALVQLFDKESSNTLYKVTILPKGPSLGHTAQLPQMDKYSYTAAEYMSNIR
AN5588.1  	LLTAYHEAGHALVAYFSP-SSMPLYKITIMPRGMSLGSTHFLPEMDMVSKNYVQYLSDID
          	 :**********:  :   *.  ***:**:*:* :** *  **:**  * .  :* . * 

MG03926.1 	SLLGGKIAEQLAFGDDMVTSGVSN0DLERATDLAYRMVSRWGMSERLGPMEYGRTFENLS
NCU03359.1	RAMGGKVAEEIVYGNELVTSGVSA0DLDMATRTAWQMVAQLGMSEKLGPVEYLRKYNQLS
FG01475.1 	VALGGKMAEELRYGGDKVTSGVSS0DLERATDLGFMMVTLFGMSATLGPVEYGRRYDNLS
AN5588.1  	VSMGGKVAEELIYGEDKVTSGISA0DLASATRTAFTLVTRFGYSKKLGNVDLYANYDSLS
          	  :***:**:: :* : ****:*  **  **  .: :*:  * *  ** ::    ::.**

MG03926.1 	SSTKAVIEAEVKTILEVAQERGAKLLKDKRVELDRLAKALVQYETLDVEEVKKVIRGEDL
NCU03359.1	SETRAMVESEVKRVLDESYERARNLLTSKRNELDYLAKALVEYETLDKKEVERVIRGEKL
FG01475.1 	SETKAAIEGEVRKTIGKSYEDVRKLLTEKRSELDLLAKALVQYETLDKSEVEKVIRGESL
AN5588.1  	SETKQEIEAEVRRLVEEARQRATNILTERRHELELLTKALIKYETLTKEEMEKVLRGESL
          	*.*:  :*.**:  :  : :   ::*..:* **: *:***::****  .*:::*:***.*

MG03926.1 	EGRTKVPENSI-LVVPKPDSDK-----PFSGGSGPPAP--LPPVPAPV--------
NCU03359.1	KDRISVPPGPMAIPKPSDTLEPGLPLPPLPGDVPPPGDSGPGPAPPPPVPA-----
FG01475.1 	PGRITAPKGPMRLPIPQQAPTP-----PGLGGVHQPQPPETPAPPAAAADTSRTDG
AN5588.1  	DRLVVPADAPMKLPEPISATNLS----PNQG-VEESGNRASAE-------------
          	      .  .: :  *      .    *  *    .
```
